# Operative Failures and Their Remedies

**Published:** 1892-12

**Authors:** Edwin C. Blaisdell

**Affiliations:** Portsmouth, N. H.


### OPERATIVE FAILURES AND THEIR REMEDIES.[1]

BY EDWIN C. BLAISDELL, D.M.D., PORTSMOUTH, N. H.

MAN is master over his method; in other words, one will produce a marked success, following a given method, where another will meet with failure, though adopting the same course, but will attain similar success with a method of his own. I am simply stating a few cases, where failures have been noticed in my practice, with perhaps a hint regarding a remedy.

First, we will consider oxyphosphate or oxychloride fillings. I seldom fill a cavity with one of these cements, without the protection of the rubber dam, producing as near as possible perfect dryness of the cavity; yet, whatever cement you use, if the cavity runs to or under the gum, it will not be uncommon for that filling to be a miserable failure, more especially if it is an approximal cavity in bicuspids or molars that are close together. As to the condition of the secretion of the gum, I am not prepared to say. Gutta-percha is not dissolved by the secretions of the mouth, whether alkaline or

---

[1] Read before the Harvard Odontological Society, September 29, 1892.

acid, and under the margin of the gum makes one of the best stoppings we have. Would it not be better, where the cavity extends under the gum, to make that portion of the filling of gutta-percha, then the remainder of cement, and in that way avoid a common failure,—viz., a large approximal cement filling inserted some two years or less, as a capping for the pulp that may have been exposed? The filling looks well, but the patient complains of pain about that tooth. You proceed to examine with a fine explorer, and find a space between the tooth and filling, below the gum. The filling is removed, the cavity is much enlarged, the pulp often exposed, and what was intended for protection has been a playground for destruction.

We have just seen where gutta-percha performs an important part in the preservation of teeth. Now, I will try and show where it in turn is destructive. For instance, a large cavity upon the grinding surface of a molar, after being excavated, little more is left than the walls of enamel standing on four sides. Several years ago I filled a number of such cavities mainly with gutta-percha, covering with amalgam to prevent wear. Many of them returned in two or three years, with one side of the enamel fractured to the gum. In two cases there was no occluding tooth; it was, therefore, hardly due to mastication. In one case the fractured enamel was replaced with amalgam, and the patient dismissed, to return in a year with more enamel gone.

I can account for this fracturing in no other way than by a slight expansion of the gutta-percha, and think that these cases call for cement for the main body of the filling. If from the close proximity of the pulp you fear harmful results from the acid in the cement, use some quick-drying varnish over the dentine.

A word for and against copper amalgam. We all know how it will waste in some mouths, also the difference in waste in regard to its location. Upon the buccal surface it is seldom affected at the gum margin, but the nearer the filling approaches the grinding surface, the greater the waste. Large copper amalgam fillings placed upon the coronal and buccal surfaces is questionable practice.

Approximal fillings of copper amalgam when protected from wear by the adjacent tooth, and not running near the grinding surface, are, in my experience, much less affected by waste.

A notice of the combination of gold and amalgam can hardly come in my paper, which treats of failures. I have not seen as much of this work as many, but what I have done and what I have seen has given satisfaction, and I have yet to see a failure. Dr.

Clapp has demonstrated this method and the success of it many times.

A few years ago I heard a member of this Society state that, for an instrument to make undercuts, "Give me a good, sharp, square-cut hoe or hatchet excavator." In the preparation of all cavities we should avoid angles as much as possible. You would never think of having a right angle in the edge of a cavity, for we are taught it is a difficult place to fill, and liable to leak and produce decay. Now, the idea of having a groove, with two right angles in it situated behind an angle, and filling it perfectly, seems beyond belief. I have tried to fill such places, and in cases where the enamel was thin I have seen, after a year or two of use, two little lines, showing the width of the instrument used in making the undercut. It is as easy to have a convex cutting edge as one with right angles, and it will make just as good an undercut, and a groove much easier to fill perfectly. A round burr I think the best. The wall that has such a groove is less liable to fracture than one made by an excavator, the cutting edge of which is square.

The most innocent-looking instrument used by the dentist is probably the matrix. Yet how many who use some of the many forms think of the danger that lies hidden in them when working gold? The closer the matrix fits the tooth, the more trouble. Here we have the same difficulty as with the undercut, only in a more dangerous position. Is it not bad practice to use one of the many forms of matrices that are held in place rigidly by any form of screw or wedge? Much more durable would the filling be if the matrix was a simple strip of watch-spring steel, held in place by the fingers, or tied in place lightly by a piece of floss silk. The good principle of such a matrix is, that when pressure is given the gold, there is a slight give to the matrix, the gold is forced and condensed to a small extent beyond the margin of the cavity; then when the cavity is filled and the matrix removed, you have a chance to do more condensing around the edges with the burnisher, which adds much life to a gold filling.

Another trouble comes to most of us in working porcelain inlays. In selecting the color for the inlay, you use great care, and find one that is very near perfection. The inlay is ground, and tried in the cavity. You congratulate yourself you have now one that even a dentist will hardly find. The inlay is then cemented into the cavity, and the cement allowed to harden. You now experience a chill down your back when you behold your presumably perfect piece of work two or three shades off color and a failure. I cannot give more than a hint towards the remedy. The trouble lies in the cement used, and will continue to remain there until some of us invent a transparent cement for such work.

The ways of working gold are many, and hardly will you find two dentists in the same office who use it in the same manner.

When we look at the work of some of our older practitioners, we find the gold fillings standing about as good as when first put in, except perhaps to the outward appearance they may look imperfect, and you judge that the fillings ought to be replaced; the old fillings are removed only to find the floor of the cavities in perfect condition. Such fillings of thirty and thirty-five years' standing are not rare. I have this summer seen six approximal fillings in the lower incisors that were inserted by Dr. E. S. Rider, of Portsmouth, in the year 1847 (forty-five years ago), and which are still protecting the cavities.

We all know that at that time soft gold was the only gold used and inserted by the wedge process. I think the philosophy of the soft gold lies in the fact that it so easily slides on itself, and takes the imprint of the wall of the cavity quickly when under side pressure.

I shall have to use the words of Dr. Gerrish, in his paper upon "Soft Gold," read before the Massachusetts Dental Society last October, when he asked, "Why does the farmer plug the tap-hole of his cider barrels with a spile made of pine, rather than some hard wood? Simply because he loses no cider, there being no leakage. For the same reason, a boat-builder stops up the holes made in his floors by the withdrawing of nails or screw, with soft wood. The plug must be softer than the material into which it is driven. When you place a soft-foil filling into a tooth, you have the same conditions present."

We all know with what extreme care a filling of cohesive gold has to be worked, what small pieces have to be used, and what the result is if there is the least rock to the first few pellets inserted.

In the use of gold there should be much judgment exercised which foil is to be selected. I think it safe to say that a cavity with four walls or more does not call for cohesive foil.

In speaking of the use of cohesive gold, I do not wish to call it a failure, for in its place there is nothing better, but we can save the teeth, and the patient and ourselves much valuable time, by using soft gold wherever possible.

There is one other point I wish to speak upon as saving time, and that is in shoeing teeth. Heretofore we have drilled two or more retaining-points or inserted screws, then built gold up or down to the desired amount. Last January I had the pleasure of seeing Dr. Levi L. Howell, of Riverhead, N. Y., give a clinic before the First District Dental Society, and through his kindness will read his method of shoeing:

"1. Drill two retaining-holes $\frac{3}{32}$ of an inch in depth, each side of the nerve, with a No. 1 round burr. Be sure that the holes are parallel.

"2. Take a piece of platina and iridium round wire, five inches long, just a trifle smaller gauge than the burr; place it in a jeweller's hand-vise, allowing the wire to protrude three (3) inches; file a sharp point on the wire.

"3. Try the wire in one hole, and see that it goes in and out easily.

"4. Take a piece of pure gold plate, No. 32 gauge; cut a piece a trifle larger than the tooth surface you wish to shoe or cap; anneal this well; place it over the tooth, and with a very sharp-pointed instrument feel for and locate the hole you have prepared your wire for; when found, just puncture the gold.

"5. Take your wire and push it to the bottom of the hole; if your wire is of right size, the gold will lock around the wire, and when it is removed the gold plate will come away with it.

"Drop a trifle of Parr's flux around the wire on the surface you are to build up.

"Take as small a piece of eighteen-karat solder as you can cut, and place it alongside of the wire on the plate; hold this over a small alcohol flame, and solder; place it on the tooth, and gently burnish the gold to its place. When you cut the first wire to free your plate, be sure and leave it projecting as far as you wish to build up.

"Place the plate on the tooth, and proceed with the second pin the same as the first. If your holes are parallel, the plate will come away attached to the wire. Solder this pin the same as the other.

"Do not cut the wire; place the plate on the tooth, and burnish until it fits perfectly. Add a thin film of gutta-percha on the side of the plate that comes in contact with the tooth; warm slightly; place the plate back, and burnish again. This gives you a perfect impression of the surface you wish to cover. Trim your plate of its overhanging edge by the marking of the gutta-percha; remove the gutta-percha, anneal, and burnish. You can now see that your plate fits every part of tooth surface.

"Drop a morsel of Parr's flux on the surface you wish to build up; place a piece of eighteen-carat solder between the two pins; hold over the lamp till the solder runs; repeat this till you have built your plate up as far as you wish. The solder will always run to the top of the first pin.

"Clean out with muriatic acid; cut your second pin off; dry your tooth, and cement in place; when set, finish with sand-paper disks, stones, files, or whatever method is suggested."

By this plan we save much pain and time, producing a shoe that will stand the wear better than the best of cohesive gold work.

